# De novo transcriptome analysis provides insights into formation of in vitro adventitious root from leaf explants of *Arnebia euchroma*

**DOI:** 10.1186/s12870-021-03172-6

**Published:** 2021-09-09

**Authors:** Jyoti Devi, Ekjot Kaur, Mohit Kumar Swarnkar, Vishal Acharya, Shashi Bhushan

**Affiliations:** 1 Biotechnology Division, CSIR-Institute of Himalayan Bioresource Technology (IHBT), Palampur,, H.P.-176061 India; 2 Academy of Scientific and Innovative Research (AcSIR), Ghaziabad-, 201002 India; 3 Dietetics & Nutrition Technology Division, CSIR-Institute of Himalayan Bioresource Technology (IHBT), Palampur,, H.P.-176061 India

**Keywords:** *Arnebia euchroma*, Adventitious roots, In vitro culture/propagation, Transcriptomics, Wounding, Auxin

## Abstract

**Background:**

Adventitious root formation is considered a major developmental step during the propagation of difficult to root plants, especially in horticultural crops. Recently, adventitious roots induced through plant tissue culture methods have also been used for production of phytochemicals such as flavonoids, anthocyanins and anthraquinones. It is rather well understood which horticultural species will easily form adventitious roots, but the factors affecting this process at molecular level or regulating the induction process in in vitro conditions are far less known. The present study was conducted to identify transcripts involved in in vitro induction and formation of adventitious roots using *Arnebia euchroma* leaves at different time points (intact leaf (control), 3 h, 12 h, 24 h, 3 d, 7 d, 10 d and 15 d). *A. euchroma* is an endangered medicinal Himalayan herb whose root contains red naphthoquinone pigments. These phytoconstituents are widely used as an herbal ingredient in Asian traditional medicine as well as natural colouring agent in food and cosmetics.

**Results:**

A total of 137.93 to 293.76 million raw reads were generated and assembled to 54,587 transcripts with average length of 1512.27 bps and N50 of 2193 bps, respectively. In addition, 50,107 differentially expressed genes were identified and found to be involved in plant hormone signal transduction, cell wall modification and wound induced mitogen activated protein kinase signalling. The data exhibited dominance of auxin responsive (AUXIN RESPONSE FACTOR8, *IAA13*, GRETCHEN HAGEN3.1) and sucrose translocation (BETA-31 FRUCTOFURANOSIDASE and MONOSACCHARIDE-SENSING protein1) genes during induction phase. In the initiation phase, the expression of LATERAL ORGAN BOUNDARIES DOMAIN16, EXPANSIN-B15, ENDOGLUCANASE25 and LEUCINE-rich repeat EXTENSION-like proteins was increased. During the expression phase, the same transcripts, with exception of LATERAL ORGAN BOUNDARIES DOMAIN16 were identified. Overall, the transcriptomic analysis revealed a similar patterns of genes, however, their expression level varied in subsequent phases of in vitro adventitious root formation in *A. euchroma.*

**Conclusion:**

The results presented here will be helpful in understanding key regulators of in vitro adventitious root development in *Arnebia* species, which may be deployed in the future for phytochemical production at a commercial scale.

**Supplementary Information:**

The online version contains supplementary material available at 10.1186/s12870-021-03172-6.

## Background

Adventitious root formation is a post-embryonic developmental process and it is considered as the most important step in vegetative propagation of woody and horticultural plant species. Adventitious rooting is not only used successfully for propagation, but it is also deployed for production of improved genotypes [1]. Adventitious roots are frequently induced from leaves, stems or hypocotyls [2]. The process of adventitious root formation can be divided into three interdependent phases: 1) induction (plant cells acquire the competence to generate founder cells), 2) initiation (formation of root primordium by divisions of the founder cells) and 3) expression or emergence phase (emergence of the newly formed adventitious roots through the epidermis) [3]. In general, cutting/wounding/detachment of tissues generate various signals at injury sites, which are then imported to converter cells (mesophyll cells, leaf margin cells and some vascular cells) [4]. Subsequently, these signals induce physiological responses such as fluctuations in plasma membrane potential, intracellular Ca^2+^ concentration, H_2_O_2_ generation and synthesis of hormones like jasmonic acid (JA) and ethylene (ET) [5, 6]. Thereafter, these responses cause fate transition of the primed competent cells (procambial, vascular parenchyma, pericycle and vascular) to founder cells that ultimately give rise to root primordia and adventitious roots [4].

In this regard, plant hormones also play a major role in different phases of adventitious root development. Primarily, auxin is the key hormone associated with all the stages of adventitious root formation and used extensively both in in vitro and in vivo conditions [5, 7]. During this process, auxin accumulates via basipetal transporter carriers in the primed founder cells of wounded plant tissues [8]. Defects in polar auxin transport inhibit the priming and division of root founder cells, thus reduces adventitious root formation [9]. Usually, auxin cause fate transition of competent cells to root founder cells by activation of WUSCHEL-related homeobox11/12 (*WOX11/12*), however, their role is not yet fully understood [10]. Auxin has been reported as a positive regulator of AUXIN RESPONSE FACTOR6 (*ARF6*) and 8 (*ARF8*) during adventitious root formation from *Arabidopsis* hypocotyls [11]. In addition, the GRETCHEN HAGEN3 (*GH3s*), *YUCCAs*, auxin efflux PIN FORMED (*PINs*), ATP binding cassette-type b (*ABCB*) and influx carrier *AUXIN1/LIKE-AUX1* genes family also play a role in adventitious root formation [12–15]. Besides auxin, cytokinin (CTK), brassinosteroids (BRs), abscisic acid (ABA), ET and gibberellic acid (GA) have also been reported to mediate adventitious roots formation [16–20]. CTK is found to maintain the root meristem during the formation of adventitious roots in apple rootstock whereas in poplar, CTK act as negative regulator of adventitious root formation [18, 21]. The process of adventitious root expression is also influenced by plant cell wall integrity. Loosening of the cell wall is an important physiological process, especially during induction and initiation phases of adventitious root formation**.** Various genes linked with cell wall modification including XYLOGLUCAN ENDOTRANSGLUCOSYLASE/HYDROLASE, PEROXIDASE, MANNITOL DEHYDROGENASE and EXTENSION-like protein are expressed during adventitious root formation [22]. Comprehensive molecular information is available for the development of adventitious roots in commercially important species in in vivo condition. The aim in most of these studies is primarily focused on improvement of propagation efficiency.

Furthermore, recently in vitro induced adventitious root cultures are used for production of phytochemicals at an industrial scale [23]. Untransformed and fast growing adventitious roots are a successful alternative to conventional full plant cultivation [24, 25]. Hence, it is pertinent to understand the underlying mechanism of adventitious root induction from detached tissues such as leaves or stems used to establish these in vitro cultures. In the present study, *A. euchroma* leaf induced adventitious roots were used for transcriptomic analysis. *A. euchroma* (Family Boraginaceae) is an endangered medicinal herb of the Himalayan region (4000–4200 m a.m.s.l). The bark of its root contains red naphthoquinone pigments, known as shikonin derivatives, which are used in Asian traditional medicine for the treatment of wounds, measles, burns and frostbite [26]. These pigments are also used as colouring agent in food, cosmetics, hair formulations and the textile industry [27]. The recent demand for these pigments led to over exploitation of *A. euchroma* plants from their natural habitat. Therefore, in vitro induced adventitious roots would be viable alternative to produce these pigments.

## Results

### Morphological observations during adventitious root development from *Arnebia* leaves

**Fig. 1:**
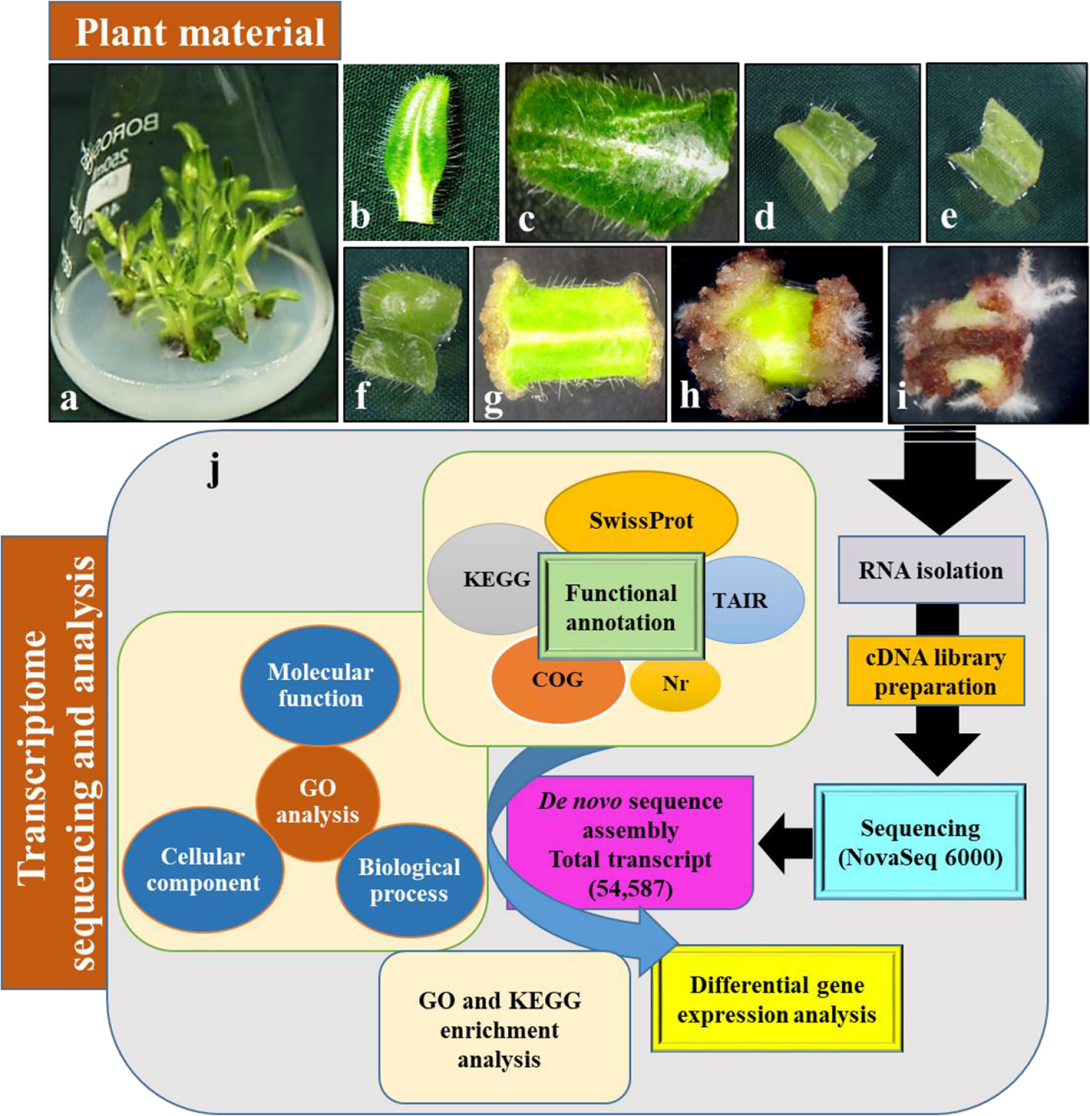
In vitro sampling and sequence of transcriptomic investigation for adventitious root formation in *A. euchroma*: **a**) shoot cultures, **b**) intact leaf and explant after inoculation in rooting media **c**) 3 h, **d**) 12 h, **e**) 24 h, **f**) 3 d, **g**) 7 d, **h**) 10 d, **i**) 15 d and **j**) transcriptomic analysis

Adventitious root formation is a post embryonic developmental process consisting of different phases (induction, initiation and expression) and each phase has its own morphology and necessities. Leaves from in vitro established shoot cultures of *A. euchroma* were used to induce adventitious roots (Fig. 1a). In brief, the leaves detached from shoot cultures were cut into small sections (4–5 mm) and inoculated on 2.5 mg/L indole-butyric acid (IBA) supplemented Schenk & Hildebrandt (SH) medium. Thereafter, it took around 3 days to show morphological changes. Explant swelling was the first sign of tissue response. However, it took around 7–10 days before explants showed induction of callus. Cytological studies revealed a dome shaped bulge, which probably is the newly initiated root primordium (Fig. 1g). After 10–15 days of inoculation, expression of adventitious roots was evident (Fig. 1h and i). Depending on these morphological observations, eight time points (3 h, 12 h, 24 h, 3 d, 7 d, 10 d and 15 d, with intact leaves as a control) were selected for analysis and divided into three phases of adventitious root formation i.e. Induction (3 h to 3 d), Initiation (3–7 d) and Expression (7–15 d) (Fig. 1b-i) to uncover genes involved in the respective phases of adventitious root formation.

### Transcriptome sequencing and assembly

mRNA libraries were prepared from tissues frozen (− 80 °C) at different time intervals of adventitious root formation from *A. euchroma* to identify the molecular mechanism of adventitious root induction. A total of 137.93 to 293.76 million raw reads was produced between different libraries (Table 1). Raw reads and used adaptor sequences were filtered using Trinity software. After trimming low quality reads and adapter sequences, 137.86 to 293.66 million high quality clean reads were obtained from the respective library with valid ratio over 99%. Transcript abundance in the respective samples was analysed via reads per kilo base of transcript, per million mapped reads (RPKM). The average RPKM in intact leaves was 18.08, whereas in samples taken from adventitious root inducing medium ranged from 10.14 to 19.72. The lowest RPKM was found in tissues taken after 15 d which showed fully developed adventitious roots (Table 1). In addition, transcript abundance showed an increased pattern from 3 h to 24 h, followed by a decrease on the 3 d and a subsequent increase on day 7 with a decrease thereafter.

**Table 1:** Summary of RNA-sequencing data on leaf induced adventitious root formation from *A. euchroma* under in vitro condition

Sample	Raw reads	Clean reads	Valid ratio%	Average RPKM
Intact leaf	248,393,724	248,308,488	99.96	18.08
3 h	137,938,400	137,868,399	99.94	18.17
12 h	178,527,526	178,457,976	99.96	18.93
24 h	181,163,274	181,053,170	99.93	19.72
3 d	293,761,606	293,661,796	99.96	19.09
7 d	24,058,770	240,518,468	99.97	19.25
10 d	264,194,742	264,111,090	99.96	18.90
15 d	175,129,672	175,059,946	99.96	10.14

Raw assembly was filtered using different levels of counts per million (CPM) [≥10, 20] and transcripts per million (TPM) [≥0.5, 1.0 & 2.0] parameters. The contiguity assessment of all the filtered assembly results revealed the finding of highest number of N50 (2193 bps) using TPM > =0.5, therefore, the total number of transcripts finally considered was 54,587 (39.4% GC) having average length of 1512.27 bps (Table 2; Supplementary Table S1).

**Table 2:** Summary of de novo assembly of *A. euchroma* leaf induced adventitious roots under in vitro condition

Quality Filtering	
Raw Reads	1,719,696,214
Filtered Reads	1,719,039,333
**TRINITY Assembly Statistics**	
No. of assembled transcripts	54,587
GC%	39.40
N50 length (bps)	2193
Average length (bps)	1512.27
No. of Predicted unigenes	31,075
GC%	39.40
N50 length (bps)	2277
Average length (bps)	1484.42

### Functional annotation and classification of unigenes with respect to adventitious root development in *A. euchroma* using GO and KEGG enrichment analysis

**Fig. 2:**
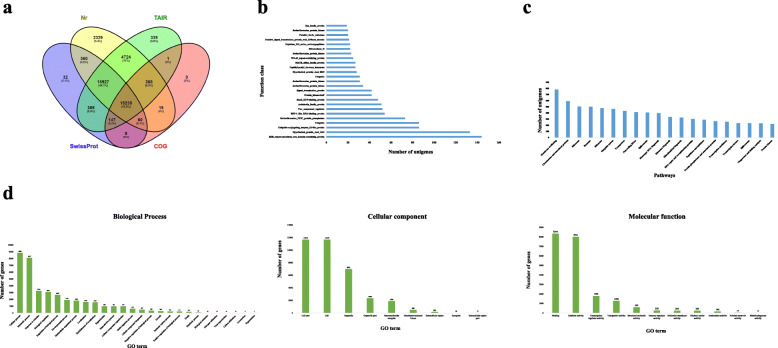
Characteristics of unigenes obtained during transcriptomic analysis: **a**) venn diagram representation of transcripts annotated by BLASTx, **b**) cluster of orthologous group classification, **c**) unigenes distribution among top 20 kyoto encyclopaedia of genes and genomes pathways and **d**) functional annotation of unigenes based on gene ontology

A number of complementary methods were used to identify the putative function of unigenes involved in adventitious rooting in *A. euchroma*. Sequence alignments of the 54,587 assembled transcripts were performed using five public databases i.e. Nr, SwissProt, COG, TAIR and KEGG using the BLASTx algorithm, with E-value <1e-05 (Supplementary Table S2). Most of the unigenes were annotated to the Nr (42,018; 76.97%) database followed by TAIR (40,106; 73.47%), SwissProt (35,126; 64.35%), KEGG (23,983; 43.93%) and COG (15,826; 28.99%) (Table 3; Fig. 2a). Thus, it is evident that Nr database is suitable for functional transcript annotation of *A. euchroma*.

**Table 3:** Summary of sequencing transcripts functionally annotated based on various public databases

Annotation database	Transcript annotated	% Annotation
Nr	42,018	76.97
SwissProt	35,126	64.35
COG	15,826	28.99
TAIR	40,106	73.47
KEGG	23,983	43.93

On the assumption that each encoded protein independently evolved from an ancestral protein, the functional classification of unigenes (54,587) was performed using COG database. Based on the number of proteins belonging to respective classes, 25 top groups were selected, whereby RHS repeat-associated core domain containing proteins (144) accounted the largest proportion followed by hypothetical protein AASI 1443 (133), UBIQUITIN-conjugating enzyme E2-like protein (86), UBIQUITIN (86) and serine/threonine PP2C protein PHOSPHATASE (73) (Fig. 2b). Furthermore, the species distribution was determined based on BLASTx annotation. Overall unigenes sequences have maximum similarity to *Sesamum indicum* (2510) followed by *Nyssa sinensis* (2474), *Handroanthus impetiginosus* (1308), *Erythranthe guttata* (558) and *Vitis vinifera* (419) (Supplementary Fig. S1). Unigenes associated with adventitious root formation in *A. euchroma* have high homology with other flowering plants.

The function of the assembled unigenes of adventitious root formation in *A. euchroma* was analysed using GO analysis (Fig. 2d). Out of the total (54,587) assembled unigenes, 42.70% were annotated into 46 functional groups and classified into cellular component, molecular function and biological processes category. The predominant biological processes were, ‘cellular process’ (8839; 37.92%), ‘metabolic process’ (8107; 34.77%), ‘response to stimulus’ (3243; 13.91%) and ‘biological regulation’ (3070; 13.16%) whereas, for ‘molecular function’ the major processes are ‘binding’ (8344; 35.79%), ‘catalytic activity’ (8011; 34.36%), ‘transcription regulator activity’ (1785; 7.65%) and ‘transporter activity’ (1268; 5.43%). For ‘cellular components’ we found ‘cell’ (11,675; 50.08%), ‘organelle’ (6971; 29.90%) and ‘organelle part’ (2336; 10.02%).

Functional enrichment was performed to decipher the metabolic processes activated during adventitious root development from leaves explant of *A. euchroma.* Organ development (GO*:* 0048513), anatomical structure development (GO: 0048856), development process (GO: 0032502) as well as response to stimulus (GO: 004222), stress (GO: 0006950), abiotic stimulus (GO: 0009628) and organic substances (GO: 0010033) were highly enriched biological processes (Supplementary Fig. S2a) and binding (GO: 0005488), transmembrane transporter activity (GO: 0022857), catalytic activity (GO: 0003824), oxidoreductase activity (GO: 0016491) and hydrolase activity (GO: 0016787) were highly identified molecular functions (Supplementary Fig. S2b). In the cellular component category, cytoplasm (GO: 0005737), organelle (GO: 0043226), membrane (GO: 0016020), mitochondrion (GO: 0005739) and nucleus (GO: 0005634) were found enriched during adventitious root formation from leaves explants of *A. euchroma* (Supplementary Fig. S2c). KEGG Automatic Annotation Server (KAAS) (https://www.genome.jp/kegg/kaas/) was also used to identify pathways significantly modulated during adventitious root formation. The analysis revealed 54,587 unigenes distributed into 428 biological pathways and the top 20 pathways are shown here (Fig. 2c).

### Identification of differentially expressed unigenes (DEGs) during adventitious root development

**Fig. 3:**
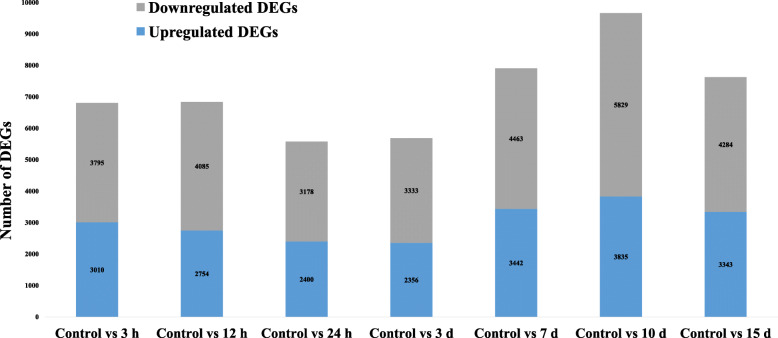
Differentially expressed genes obtained from RNA-sequencing during different phases of adventitious root formation in *A. euchroma*

DEGs during in vitro adventitious root formation from leaves explants of *A. euchroma* were identified using IDEG6 program at the respective time points i.e. 3 h, 12 h, 24 h, 3 d, 7 d, 10 d and 15 d (Fig. 3) (Supplementary Table S3a & b). The individual sampling time was compared with intact leaves, which served as control. Genes with expression levels with fold change (FC) ≥1.0 and *p*-value≤0.001 were considered upregulated, whereas genes with ≤1.0 FC were downregulated. In total, 50,107 DEGs were observed at different stages of adventitious root formation, out of which 21,140 transcripts were upregulated and 28,967 downregulated. De novo DEG analysis revealed 6805, 6839, 5578, 5689, 7905, 9664 and 7627 DEGs at selected seven time points during adventitious root formation. The number of upregulated DEGs was highest on the 10th day as compared to another adventitious root formation stages. Overall, the number of upregulated genes in the present study was lower than the number of downregulated genes. After inoculation of leaves explant, the number of upregulated DEGs were found to be decreased from 3 h to 3 d and later increased up to day 10 of adventitious root formation. The 3rd day expression levels seemed to be a central point in adventitious root formation from which number of DEGs changed. However, with clear expression of adventitious roots after 15th day of inoculation, the number of upregulated DEGs decreased. After downstream analysis of DEGs obtained from present study, numerous transcripts were found to be involved in phytohormones mediated signalling. These include auxin-responsive [*IAA10* (DN393_c0_g1_i1), *IAA6* (DN6765_c0_g2_i1), *IAA13* (DN11362_c0_g5_i1), *IAA18* (DN19740_c0_g1_i4), *ARF8* (DN3_c1_g3_i3), *ARF18* (DN7598_c0_g1_i4), *ARF9* (DN3003_c0_g1_i4), *GH3.1* (DN18251_c0_g1_i2) and *GH3.6* (DN3834_c0_g1_i1)], CTK [*CRE1* (DN3714_c0_g1_i15), *ARABIDOPSIS THALIANA* HISTIDINE PHOSPHOTRANSFERASE (*AHP*) (DN12424_c0_g1_i2), type-B ARABIDOPSIS RESPONSE REGULATORS (*B-ARR*) (DN1969_c0_g2_i3) and ISOPENTENYL TRANSFERASES5 (*IPT5)* (DN7996_c0_g1_i3)] and GA [*GIBBERELLIN-INSENSITIVE DWARF2* (*GID2*) (DN12152_c0_g1_i1), DELLA protein, RalGDS-like2 protein (*RGL2*) (DN8804_c0_g2_i3), PHYTOCHROME-INTERACTING FACTOR 3 (*PIF3*) (DN4153_c0_g1_i6)] signalling components. In addition, the genes related to BRs [BRASSINOSTEROID INSENSITIVE1-associated receptor KINASE1 (*BAK1*) (DN3660_c0_g2_i1), BRASSINOSTEROID INSENSITIVE1 (*BRI1*) (DN425_c0_g2_i1; DN893_c1_g1_i2) *TCH4* (DN19545_c0_g1_i1; DN1414_c0_g1_i1; DN10732_c0_g1_i3)] and phenylalanine metabolism [*TGA* (DN29918_c1_g1_i4), PATHOGENESIS-RELATED protein1 (*PR1*) (DN95671_c0_g1_i1)] were also found expressed during the adventitious root formation process. Beside these transcripts, DEGs related to auxin interaction with JA [*TIF9* (DN13128_c0_g1_i3) and *MYC2* (DN2592_c0_g1_i6)] and *ERF114* (DN3647_c0_g3_i1) were also expressed during this process.

**Fig. 4:**
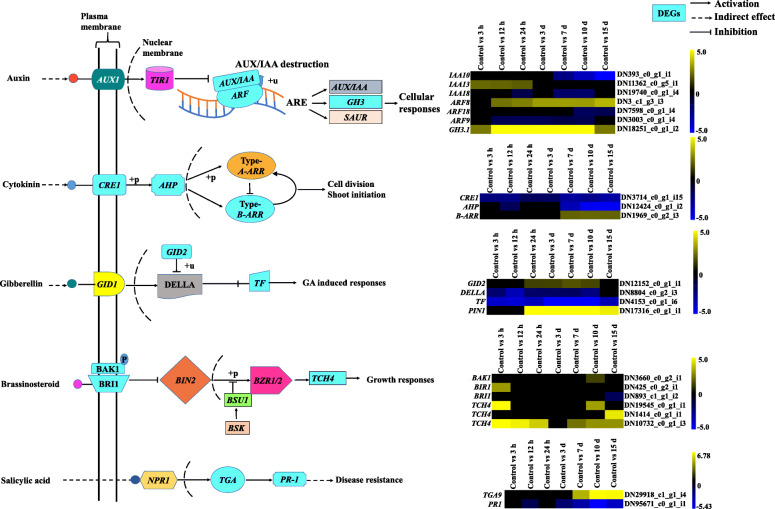
Relative expression (fold change 1.0) of unigenes involved in key pathways of adventitious root formation (control vs 3 h, 12 h, 24 h, 3 d, 7 d, 10 d and 15 d); +u: ubiquitination, +p: phosphorylation [32]

Based on the results, *IAA13* (DN11362_c0_g5_i1) was upregulated during initial stages (3 h to 24 h), whereas *ARF8* (DN3_c1_g3_i3) from 12 h to 15th day and *GH3.1* (DN18251_c0_g1_i2) throughout the adventitious root formation. Moreover, *ARF9* (DN3003_c0_g1_i4) showed continuous downregulation from 12 h to 10th day. In case of CTK signalling, the differential expression of *CRE1* (DN3714_c0_g1_i15) and *AHP* (DN12424_c0_g1_i2) phosphorelay mediator was also found downregulated during the course of adventitious root development (Fig. 4). Interestingly, *B-ARR* (DN1969_c0_g2_i3) showed maximum upregulation after 10th days of leaf explant inoculation. Genes associated with GA signalling i.e. *DELLA* (DN8804_c0_g2_i3) and *PIF3* (DN4153_c0_g1_i6) showed downregulation throughout the formation of adventitious roots. However, *GID2* (DN12152_c0_g1_i1) was upregulated from 24 h (induction phase) to 7th day (initiation phase) and then showed a sudden decreased expression pattern. The expression profile of BRs associated genes was also examined among DEGs. The gene *TCH4* (DN10732_c0_g1_i3) was upregulated throughout the formation of adventitious root except at 3 h of inoculation of leaves explants. In addition, a gene of phenylalanine signalling pathway i.e. *PR1* (DN95671_c0_g1_i1) encoding a pathogen related protein was downregulated during the different phases of adventitious root formation.

Besides phytohormones, genes related to wound mitogen-activated protein kinase (MAPK) signalling were also found differentially regulated. These include CALMODULIN-4 (*CAM4*) (DN10034_c0_g2_i2), MITOGEN-ACTIVATED PROTEIN KINASE KINASE3 (*MKK3*) (DN3958_c0_g1_i4), MITOGEN-ACTIVATED PROTEIN KINASE8 (*MPK8*) (DN1916_c0_g1_i4), RESPIRATORY BURST OXIDASE HOMOLOG PROTEIN C (*RBOHC*) (DN1706_c0_g1_i1) and OXIDATIVE STRESS INDUCIBLE1 (*OXI1*) (DN5508_c0_g1_i11). Out of these, *CAM4* (DN10034_c0_g2_i2) showed higher expression after 3d of inoculation, whereas *MPK8* (DN1916_c0_g1_i4) expressed quite early i.e. after 3 h. Furthermore, *MKK3* (DN3958_c0_g1_i4) was upregulated during the expression phase (7–15 d) and *OXI1* (DN5508_c0_g1_i11) showed downregulation for all time points. In addition to the genes highlighted above, two genes associated with sink establishment were also expressed during the formation of adventitious roots. These genes were APOPLASTIC INVERTASE/CELL WALL INVERTASE or BETA-FRUCTOFURANOSIDASE (DN6157_c0_g2_i2) and MONOSACCHARIDE TRANSPORTER or MONOSACCHARIDE-SENSING protein2 (DN25_c0_g1_i6), which get expressed after 3 h (early induction phase) and later downregulated. LATERAL ORGAN BOUNDARIES DOMAIN16 (*LBD16*) (DN2020_c0_g1_i1) showed higher expression after 7 days of leaf explant inoculation.

In case of cell wall modification proteins, EXPANSIN-B15 (DN144245_c0_g1_i1), PECTINESTERASE INHIBITOR7 (DN169482_c0_g1_i1), ENDOGLUCANASE25 (DN27221_c0_g1_i1), LEUCINE-rich repeat EXTENSIN-like protein2 (DN18307_c0_g1_i1) and XYLOGLUCAN ENDOTRANSGLUCOSYLASE/HYDROLASE protein4 (DN28324_c0_g1_i5) were expressed in the wounding tissue. Out of these, XYLOGLUCAN ENDOTRANSGLUCOSYLASE/HYDROLASE protein4 (DN28324_c0_g1_i5) showed upregulation from 3 to 24 h of inoculation, whereas PECTINESTERASE INHIBITOR7 (DN169482_c0_g1_i1) and LEUCINE-rich repeat EXTENSIN-like protein2 (DN18307_c0_g1_i1) 24 h onwards.

## Discussion

*Arnebia euchroma*, a traditional plant of the Himalayan region, is well known for its medicinal properties as well as its colouring pigments. The high demand of plants collected from wild, especially after the covid-19 pandemic, necessitate in vitro technology like tissue culture as an alternative source to extract phytochemicals. Therefore, we tried to induce adventitious root from leaves explants of *A. euchroma*. Efforts were made to understand the adventitious root induction process and samples were collected from different adventitious root formation stages to carry out a transcriptomic study. In this study, transcriptomic changes observed during adventitious root formation in in vitro conditions were reported.

### Determination of unigenes during adventitious root development in *A. euchroma*

Leaves tissues removed from the mother plant experience changes that cause alterations in gene expression during wounding and subsequent root formation. To understand such phenomena, Illumina RNA-seq technique is used extensively for transcriptomic study with or without reference information/data. The present study was performed using NovaSeq 6000 platform in order to understand the expression of genes during in vitro adventitious root formation in *A. euchroma*. It is necessary to decipher the molecular processes associated with adventitious rooting and identify transcripts that can be used for manipulation of their growth and phytochemical production under in vitro conditions.

### Plant hormones in adventitious root formation

Plant hormones play an important role during different developmental stages i.e. induction, initiation and expression of adventitious root formation either individually or in combination [5]. One of the major hormone is auxin that initiates the adventitious roots from wounded tissue. In addition to endogenous auxin, there are number of reports that showed its external application for adventitious root formation [7, 28]. Likewise, such roots can also be induced from detached leaves without exogenous application of auxin as reported in *Arabidopsis* [17]. The type and exposure time to auxin also effect the formation of adventitious roots [29]. Auxin also acts as mediator of developmental processes by interacting with other plant hormones like CTK, ET, JA, strigolactones and BRs. For example, the development of primary root and root hair growth is promoted by auxin and ET synergistically, however during lateral root formation their effect is antagonistic [30]. Furthermore, higher auxin concentrations promote adventitious root induction, but play a repressive role during later stages (initiation and expression) [31]. However, until now there is no study in which adventitious root formation in the medicinal plant *A. euchroma* is reported. Therefore, we made efforts to understand the formation process of in vitro adventitious roots in *A. euchroma.*

In the present study, among the DEGs, phytohormones-related genes were identified. These included transcriptional controls of auxin, CTK, GA, BRs biosynthesis, JA and SA signalling (Fig. 4) [32]. Four auxin responsive genes (*IAA6*, *10*, *13* and *18*) were differentially expressed, which indirectly endows to auxin homeostasis during in vitro adventitious root formation. *IAA6* (DN6765_c0_g2_i1) and *IAA13* (DN11362_c0_g5_i1) showed the highest expression during early induction phase. Li et al. (2018) also reported the high expression level of *IAA29* during early phase of adventitious root formation in apple stem cuttings with subsequent decrease in later periods [7]. A recent study on de novo root regeneration from leaf explants of *Arabidopsis* mutants revealed a specific role of *IAA14*, *IAA18* and *IAA28* during vascular proliferation and root initiation [33]. *ARF8* (DN3_c1_g3_i3) showed a positive role for adventitious root formation due to increased expression during induction (12 h to 3 d) and expression phase (10 to 15 d). Similar findings were reported in *Arabidopsis*, where they found *ARF8* a positive regulator of adventitious root formation [11]. *GH3.1* (DN18251_c0_g1_i2) was significantly upregulated and showed the highest expression during early induction phase (12 h). In *A. thaliana*, *GH3* (*GH3.3*, *GH3.5* and *GH3.6*) expression was also found stimulated by auxin during adventitious roots formation from the hypocotyl [12]. These and our results are inconsistence with a study in apple rootstock, wherein the expression of *GH3.1* was found highest after 3 days of inoculation in rooting medium [7].

Beside auxin, CTK also has a role in adventitious root formation [34]. CTK positively controls cell division and shoots development. In *Arabidopsis*, CTK acts as inhibitor of auxin and interrupts PIN genes expression in root founder cells, thereby averting the auxin gradient required for patterning of root primordia [35]. In addition to this, *ptRR13*, a *Populus* type-b response regulator of CTK acts downstream and represses adventitious root formation [18]. In the current study, *CRE1* (DN3714_c0_g1_i15) encoding a membrane-localized receptor of CTK signalling and *AHP* (DN12424_c0_g1_i2) were identified and downregulated throughout the development of adventitious roots. Furthermore, *B-ARR* (DN1969_c0_g2_i3), a two component response regulator like protein was found upregulated during expression phase, indicating that CTK may be involved in the final steps of adventitious root development in *A. euchroma.*

GA controls various aspects of plant growth such as seed germination, reproductive growth, cell elongation and flowering, and generally inhibits adventitious root formation, but has a positive effect on root elongation [36]. It interrupts polar auxin transport through DELLA proteins, thereby competent cells are unable to form the auxin maxima required for their fate transition to founder cells during adventitious root formation [37, 38]. DELLA proteins, transcriptional factor of the GAI-RGA-AND-SCR (GRAS) family, are negative regulators of GA signalling [37]. Ensuing GA binding, GID1 receptor induces DELLA degradation via *Arabidopsis* SCF^SLY1^ or SCF^SNE^ complexes [39–42]. There are reports that revealed that DELLA proteins block auxin transport in root tips through the GA-stimulated deprivation of well-known PIN1 and PIN2 transporters [43, 44], thus it’s degradation is required for normal GA signalling and PIN proteins accumulation. In present work, four GA signalling related DEGs were detected. Among these DEGs, *GID2* (DN12152_c0_g1_i1) and *PIN1* (DN17316_c0_g1_i1) showed upregulation, whereas *DELLA* (DN8804_c0_g2_i3) and TRANSCRIPTIONAL FACTOR (*TF*) (DN4153_c0_g1_i6) were downregulated during all the phases of adventitious root development. Considering these findings, the persistent downregulation of DELLA in present study probably led to the expression of *PIN1* (DN17316_c0_g1_i1). Hence, this indicates that auxin crosstalks with GA during adventitious root development. In contrast, GAs was also found to repress adventitious root formation in *Popular* and *Arabidopsis* by disturbing transport of auxin [45]. So, the likely profound presence of GA-auxin related genes might have a crucial role in adventitious root development in *A. euchroma.*

In addition, auxin-BR interactions are also essential for adventitious root formation. These hormones are found to control cell expansion, proliferation, vascular differentiation and root growth [46]. In *Arabidopsis*, lateral root initiation is affected by BRs in an auxin transport dependent manner [46]. In the present investigation, *BRI1* (DN425_c0_g2_i1) showed differential expression after 3 h (early induction phase) and seems to interact with *BAK1* (DN3660_c0_g2_i1) receptor (10 d) to form the BRI1/BAK1 complex. This complex is known to inhibit GLYCOGEN SYNTHASE KINASE3 (*GSK3*)/SHAGGY, which in turn activates BRs responsive gene i.e. BRASSINAZOLE-RESISTANT1 (*BZR1*). In addition to this, *TCH4* (DN10732_c0_g1_i3), a downstream regulator of BRs signalling showed highest expression after 3 h (early induction phase) and a subsequent decrease thereafter [47].

**Fig. 5:**
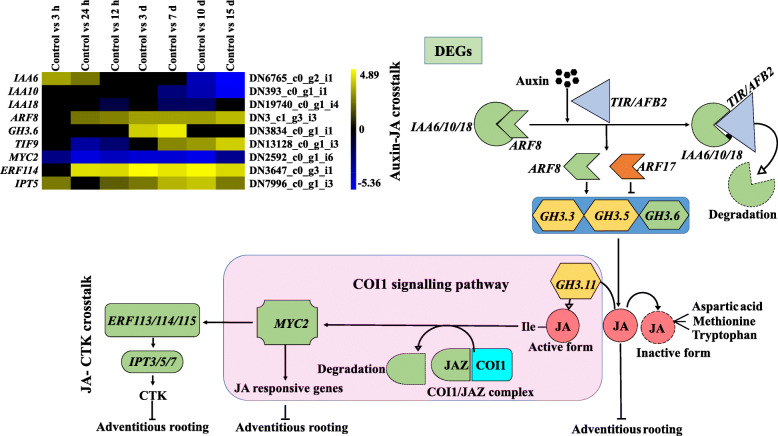
Interaction of auxin with jasmonic acid (JA) and cytokinin (CTK) during formation of *A. euchroma* adventitious roots under in vitro condition [32]

Furthermore, interaction of auxin with JA and CTK during formation of adventitious roots was also evident in the present study [32]. Among various TFs, *ARF8* (DN3_c1_g3_i3) and *GH3.6* (DN3834_c0_g1_i1) were found to be differentially expressed. *ARF8* (DN3_c1_g3_i3) expression was upregulated in all the three adventitious root formation phases, whereas *GH3.6* (DN3834_c0_g1_i1) only showed higher expression in initiation phase. In *Arabidopsis*, proteasome-mediated breakdown of *IAA6*/*10*/*18* repressor by TRANSPORT INHIBITOR RESPONSE1/AUXIN-SIGNALLING F-BOX2 (*TIR1/AFB2*) auxin receptor promotes transcriptional activity of *ARFs* that acts upstream of the JA signalling pathway [48]. These *ARFs* are reported to upregulate the expression of *GH3.3*, *GH3.5* and *GH3.6* proteins that encode ACYL-ACID-AMIDO SYNTHETASES [12]. In *Arabidopsis*, these enzymes were found to be responsible for the amino acid conjugation of JA, a known inhibitor for the formation of adventitious roots [12]. JA is known to inhibit cell division by repressing the transcription of B-type CYCLIN dependent KINASES [49]. As discussed earlier, transcriptional repression of *IAA6*/*10*/*18* is responsible for upregulated expression of *ARF8* (DN3_c1_g3_i3), that subsequently activates *GH3.6* (DN3834_c0_g1_i1). Probably, this activation led to JA-tryptophan inactive complex formation by conjugation (Fig. 5). This might trigger the expression of cell division related genes leading to the formation of adventitious roots. With regards to JA signalling pathway, *TIF9* (DN13128_c0_g1_i3) a JASMONATE ZIM DOMAIN (*JAZ*) co receptor and *MYC2* (DN2592_c0_g1_i6) were found differentially expressed under different phases of adventitious root formation in present investigation. *TIF9* (transcriptional repressor) was upregulated during initiation and expression phase, whereas *MYC2* (transcriptional activator) was found downregulated throughout the adventitious root formation process in *A. euchroma*. These transcriptional factors i.e. *TIF9* and *MYC2* are the known regulators of JA dependent genes [50]. It is evident that upregulation of *TIF9* might be responsible for lowering the expression of *MYC2* that in turn inhibit JA responsive genes. In accordance to present results, the upregulated expression of *JAZ12* and *26* was reported in initiation and expression phase of adventitious root development in apple cuttings [7]. Similarly, *MYC2* was revealed to be downregulated during adventitious roots induction in *Arabidopsis* hypocotyls [12]. Moreover, *MYC2* also regulate the expression of mechanical wounding induced ETHYLENE RESPONSE FACTORs (*ERFs*) [51, 52]. These factors are known to act downstream of the JA signalling pathway and activate the expression of *IPT 3/5/7*, which plays a significant role in CTK biosynthesis [53]. Largely, *ERF114* (DN3647_c0_g3_i1) and *IPT5* (DN7996_c0_g1_i3) observed to be upregulated in all the phases, except 3 h and 12 h, respectively (Fig. 5) [32]. It can thus be concluded that JA and CTK related genes possibly interact with auxin signalling during the formation of adventitious roots in *A. euchroma*.

Overall, different phytohormones form a complex network that interacts at various levels to influence adventitious root formation in in vitro conditions. It can be inferred from the above results and discussion that differential expressions of genes related to auxin, CTK, GAs, BRs and JA signalling plays a significant role in adventitious root formation in *A. euchroma.*

### Wounding related MAPK signalling pathway

Wounding caused by mechanical damage, herbivore or insect attack produces various signals that initiate a number of physiological process. However, the mode of action of these signals in plant regeneration as well as healing of damaged tissue or defence is not fully explored [54]. After wounding, the cytosolic Ca^2+^ increases quickly that stimulate *CAMs*, i.e. Ca^2+^ binding proteins known to mediate intercellular Ca^2+^ pathways. This leads to the formation of Ca^2+^/CAM complexes, which play an important role in reactive oxygen species (ROS) scavenging [55].

**Fig. 6:**
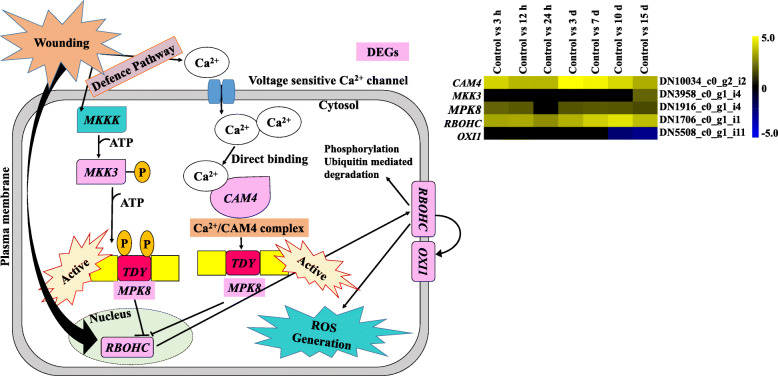
MAPK signalling transcripts identified during wound induced adventitious root formation in *A. euchroma* [32]

Similarly, five DEGs related to wound MAPK signalling pathway were discovered in the present study (Fig. 6) [32]. These include *CAM4* (DN10034_c0_g2_i2), *MPK8* (DN1916_c0_g1_i4), *MKK3* (DN3958_c0_g1_i4) and *RBOHC* (DN1706_c0_g1_i1). *CAM4* showed higher expression after 3 days (induction phase), while *MPK8*, *MKK3* and *RBOHC* get expressed during expression phase. MPK8, a protein kinase, gets activated either by *CAM4* or *MKK3* independently that regulates the expression of *RBOHC* and ROS homeostasis triggered by wounding [55]. In the present study, *RBOHC* (DN1706_c0_g1_i1) observed to be expressed after 10 d (expression phase), it is thus evident that wound induced signals play crucial roles in adventitious root formation in *A. euchroma*.

### Cell wall modification

**Fig. 7:**
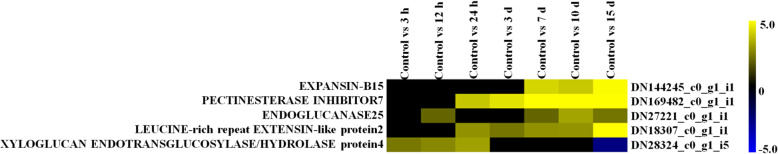
Heat map depicting differentially expressed plant cell wall modification genes during in vitro adventitious root formation in *A. euchroma*

The cell wall has direct impact on growth and development of any plant tissue. It also plays a critical role in defence mechanism against wounding or invasion by pathogens [56]. In general, the wounding causes mechanical damage to the cell wall and generate multiple signals that led to change in plasma membrane potential, intracellular Ca^2+^ concentration and production of H_2_O_2_ including hormone like JA and ET [38]. Similarly, wounding of tissue during induction of adventitious root caused shift in cell wall dynamics and results in its softening probably required for emergence of the newly formed adventitious roots. In the present investigation, five genes i.e. EXPANSIN B-15 (DN144245_c0_g1_i1), PECTINESTERASE INHIBITOR7 (DN169482_c0_g1_i1), ENDOGLUCANASE25 (DN27221_c0_g1_i1), LEUCINE-rich repeat EXTENSION like protein2 (DN18307_c0_g1_i1) and XYLOGLUCAN ENDOTRANSGLUCOSYLASE/HYDROLASE protein4 (DN28324_c0_g1_i5) were found to be differentially expressed (Fig. 7). EXPANSIN B-15 (DN144245_c0_g1_i1) was differentially expressed during late initiation and expression phase of adventitious root formation. This expansin is reported to be expressed in fast growing tissues and disrupt hydrogen bonds between cellulose micro fibrils, which results in loosening of the cell wall [57]. These genes were reported to play a role in adventitious root development in the model plant *Arabidopsis* [58].

### Other regulators involved in adventitious root formation

*LBD16/18* play important roles in wound-induced, hypocotyl-induced adventitious root formation in *Arabidopsis* [59]. In present study, *LBD16* (DN2020_c0_g1_i1) was highly upregulated during the initiation phase (7 d). Furthermore, BETA-FRUCTOFURANOSIDASE/ CELL WALL INVERTASE (DN6157_c0_g2_i2) and MONOSACCHARIDE TRANSPORTER/ MONOSACCHARIDE-SENSING protein1 (DN25_c0_g1_i6) were differentially expressed during early induction phase, but afterwards showed decreased expression in the subsequent phases of adventitious root formation. BETA-FRUCTOFURANOSIDASE also known as CELL WALL INVERTASE (EC 3.2.1.26), is responsible for hydrolysis of sucrose into glucose and fructose and required for carbon metabolism, storage and transport [60]. Whereas, MONOSACCHARIDE TRANSPORTERS involved in translocation of these hydrolysed products to the wounded tissues. Thus both are critical for adventitious root formation in *A. euchroma*. Similarly, Ahkami et al. (2014) reported these two proteins in adventitious root induction from *Petunia* cuttings [61].

## Conclusion

**Fig. 8:**
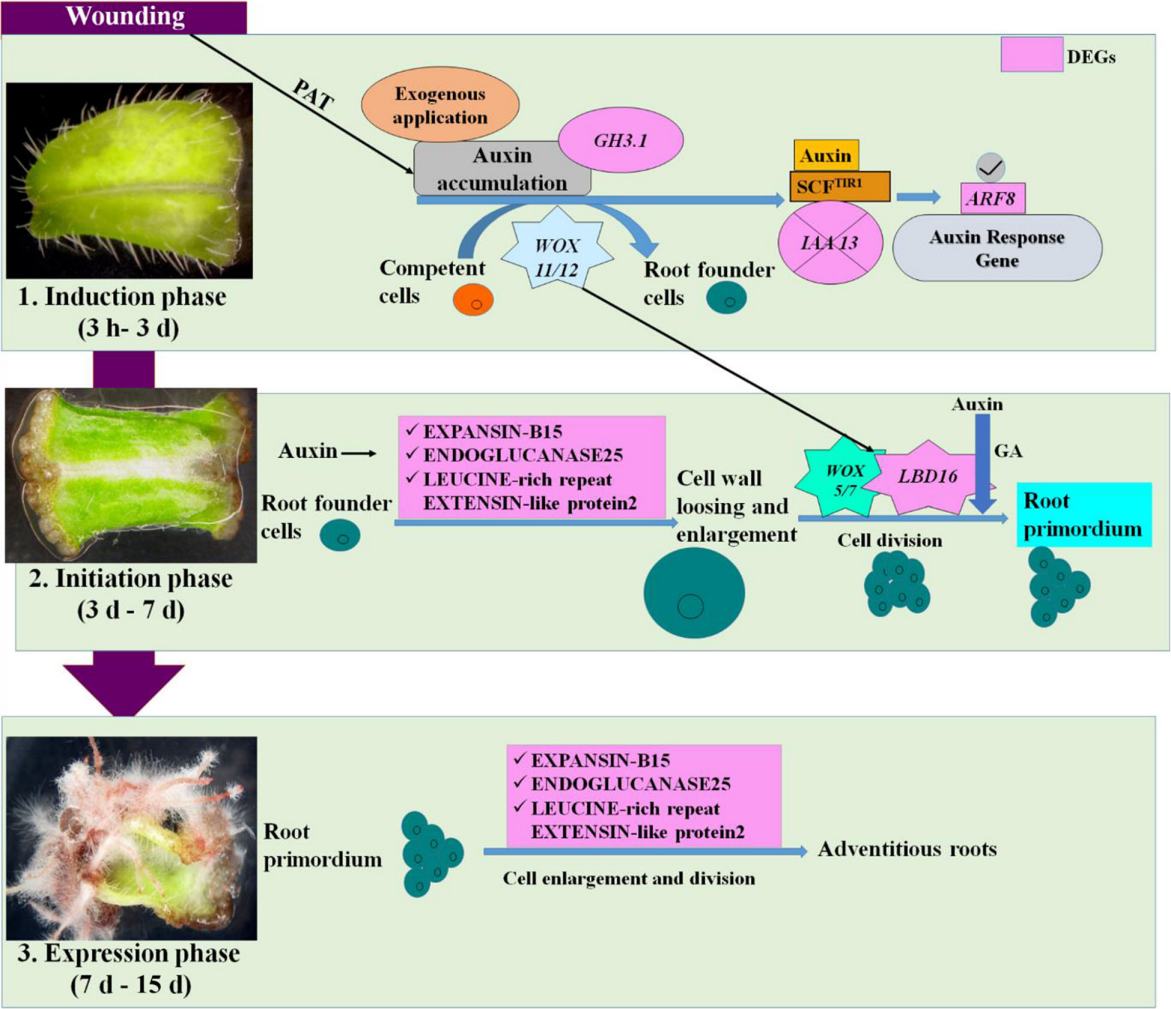
Proposed underlying mechanism for leaf induced adventitious root development from *A. euchroma* under in vitro conditions

Considering the potential of in vitro induced adventitious roots as an alternative source of phytochemicals, it is necessary to understand their induction mechanism and identify factors that are critical for efficient process development. With this regard, transcriptomic analysis was performed to apprehend the formation of leaf induced adventitious roots from *A. euchroma*. The study revealed three physiologically distinct phases of adventitious root formation i.e. induction, initiation and expression. The findings of the present investigation revealed the role of plant hormone signalling pathways in adventitious root formation, however, the contribution of individual genes needs to be substantiated before they can be applied in commercial in vitro systems. A hypothetical model of adventitious root formation based on our results is shown in Fig. 8.

## Methods

### Plant material and in vitro induction of adventitious root from *Arnebia euchroma*

*Arnebia euchroma* plants were collected from Kibber (32.33 °N, 78.00 °E), Spiti valley, Himachal Pradesh, India and maintained under polyhouse conditions (annual temperature range: 20–30 °C; Relative humidity 60–70%) at CSIR-IHBT Palampur. The formal identification of the plant material used in the current study was done by Dr. Shashi Bhushan, CSIR-IHBT Palampur. The plant sample is preserved in the institutional herbarium vide voucher specimen no PLP18571. Young rhizome buds of healthy plants were rinsed with distilled water to remove the dust particles and brushed with Tween-20 for 7–8 min. After that these buds were surface sterilized using 0.05% (w/v) bavistin and streptomycin sulphate (0.05% w/v) for 8–10 min followed by mercuric chloride (0.02%, w/v) as described previously [62]. Surface sterilized, buds were then inoculated vertically in Murashige and Skoog medium augmented with kinetin (1.0 mg/L). Medium was sterilized at 121 °C temperature for 15 min. These cultures were then incubated under 16/8 h’ photoperiod in an aseptic culture room having constant temperature (25 ± 2 °C). After 1 month of inoculation buds open-up and proliferated into shoot. *A. euchroma* leaves from these aseptic shoots were detached, cut into small sections (4–5 mm) and inoculated on SH medium supplemented with 2.5 mg/L IBA, 3% sucrose and 0.25% CleriGel (HiMedia) for adventitious roots induction. For transcriptomic analysis, four explants per petri plate with two replicates were sampled at respective intervals i.e. after 3 h, 12 h, 24 h, 3 d, 7 d, 10 d and 15 d. The samples were then instantly freezed in liquid nitrogen and stowed at − 80 °C prior to RNA isolation. Intact leaf removed from the shoot was immediately freezed that served as control for the experiment.

### Total RNA isolation, mRNA library preparation and sequencing

The total RNA was isolated by iRIS method developed previously at host institute [63]. Total RNA was then subjected for quality and quantity assessment using nanodrop 1000 spectrophotometer (Thermo Fisher Scientific, USA) and Bioanalyzer RNA nano chip (Agilent 2100 Technologies, USA) respectively. Two independent biological replicates were taken for further experiments. High quality total RNA (5.0 μg) of each replicate was used for cDNA library preparation using TruSeq mRNA standard sample Prep Kit v2 (Illumina Inc., USA) following the manufacturer’s instructions. Purified cDNA libraries were then quantified via a fluorescence based quantification system (Qubit, Life Technologies, USA) using a dsDNA HS assay kit. The library insert size of about 260 bps was confirmed using Bioanalyzer DNA 1000 chip. A total of sixteen independent paired-end libraries were prepared, followed by denaturation and loaded at 400 pM for high throughput DNA sequencing using the NovaSeq 6000 platform.

### Data processing and de novo assembly

Adapter sequences used for cDNA library preparation and raw reads generated by NovaSeq 6000 platform sequencing were subjected to quality evaluation as per default trimmomatic parameters to filter out adapter and low-quality bases. In addition, the quality of reads also enhanced by discarding short contigs of < 200 bps. The reference assembly was obtained by concatenating the high-quality clean reads with k-mer length of 25 bps of eight samples using Trinity program (https://github.com/trinityrnaseq/trinityrnaseq/wiki) [64]. To predict open reading frame from the leftover transcripts, Trans Decoder (http://transdecoder.github.io) was carried out for identification of coding sequence regions. These assembled sequences were called unigenes and subjected to cluster database at high identity with tolerance (CD-HIT) program (https://github.com/weizhongli/cdhit) at 95% identity (−c 0.95 –T 8 –M 2000–gap − 2) to acquire non-redundant unigenes by discarding repeated sequences which were further subjected to annotation and functional analysis. The transcriptome profiling data thus is submitted for public use in NCBI SRA database (Accession number PRJNA695864).

### Computational annotation of putative transcripts

The collected unigenes were examined by BLASTx algorithm against different databases using E-value parameters set to 1e-05. The databases included SwissProt (https://www.uniprot.org), KEGG (https://www.genome.jp/kegg), COG (https://www.ncbi.nlm.nih.gov/research/cog/), Nr (https://ftp.ncbi.nlm.nih.gov/blast/db) and TAIR (https://www.arabidopsis.org/download). GO of total transcripts was performed using agriGO to ascertain its functional annotation [65]. In addition, the pathway enrichment analysis of assembled unigenes was also analysed by KAAS (https://www.genome.jp/kegg/kaas/) [66]. The overall information on databases used in present study for transcriptomic analysis is depicted in Fig. 1j.

### *In-silico* analysis of differentially expressed transcripts

The expression level of each unigene obtained from leaf explants of *A. euchroma* was compared with control at different time interval and analysed via Trinity software. In de novo transcriptome analysis, abundance was predicted and normalized via RNA-Seq by expectation maximization (RSEM) package (http://deweylab.biostat.wisc.edu/rsem/). Differential expression of transcripts was performed using edgeR package [67] in intact leaf vs 3 h, 12 h, 24 h, 3 d, 7 d, 10 d and 15 d of leaf explant inoculation. The critical selection criteria to identify DEGs was set to the following parameters: Log FC ≥ 1.0 and *p-*value ≤ 0.001 for upregulated and FC ≤ 1.0 and *p*-value ≤ 0.001 for downregulated DEGs.

## Supplementary Information

**Additional file 1 Fig. S1.** The species distribution of unigenes BLASTx results.

**Additional file 2 Fig. S2.** Functional annotation with respect to adventitious root development in *A. euchroma* using GO enrichment analysis.

**Additional file 3 Table S1**. Summary of de novo assembly of *Arnebia euchroma* leaf induced adventitious roots under in vitro conditions.

**Additional file 4 Table S2**. Summary of BLASTx results for the *A. euchroma* transcriptome against five databases.

**Additional file 5 Table S3a**. List of differentially expressed genes, fold change ≥ 1.0. **Table S3b.** List of differentially expressed genes, fold change ≤ 1.0.

## Data Availability

The transcriptome profiling data is submitted in NCBI SRA database under the BioProject PRJNA695864 (https://dataview.ncbi.nlm.nih.gov/object/PRJNA695864?reviewer=2ev0m5a9ekgim1293n69cmq72d). All datasets used are open-sourced and links of the same cited appropriately in the manuscript.

## Abbreviations

*A. euchroma*: *Arnebia euchroma*
h: Hours
d: Days
bps: Base pairs
JA: Jasmonic acid
ET: Ethylene
*WOX11/12*: WUSCHEL-RELATED HOME BOX11/12
*ARF6*: AUXIN RESPONSE FACTOR6
*ARF8*: AUXIN RESPONSE FACTOR8
*GH3*: GRETCHEN HAGEN3
*PIN*: PIN FORMED
*ABCB*: ATP BINDING CASSETTE-type B
CTK: Cytokinin
BRs: Brassinosteroids
ABA: Abscisic acid
GA: Gibberellic acid
IBA: Indole-3-butyric acid
SH: Schenk & Hildebrandt
CPM: Counts per million
TPM: Transcripts per million
RPKM: Reads per kilo base of transcript, per million mapped reads
GO: Gene ontology
KEGG: Kyoto encyclopaedia of genes and genomes
Nr: NCBI non-redundant database
COG: Clusters of orthologous groups
TAIR: The Arabidopsis information resource databases
KAAS: KEGG Automatic Annotation Server
DEGs: Differentially expressed unigenes
*AHP*: *ARABIDOPSIS THALIANA* HISTIDINE PHOSPHOTRANSFERASE
*B-ARR*: Type-B ARABIDOPSIS RESPONSE REGULATORS
*GID2*: GIBBERELLIN-INSENSITIVE DWARF2
*RGL*: RalGDS-like2 protein
*PIF3*: PHYTOCHROME-INTERACTING FACTOR3
*BAK1*: BRASSINOSTEROID INSENSITIVE 1- associated receptor KINASE1
*BRI1*: BRASSINOSTEROID INSENSITIVE1
*PR1*: PATHOGENESIS-RELATED PROTEIN1
*MAPK*: MITOGEN-ACTIVATED PROTEIN KINASE
*CAM4*: CALMODULIN4
*MKK3*: MITOGEN-ACTIVATED PROTEIN KINASE KINASE3
*MPK8*: MITOGEN-ACTIVATED PROTEIN KINASE
*RBOHC*: RESPIRATORY BURST OXIDASE HOMOLOG PROTEIN C
*OXI1*: OXIDATIVE STRESS INDUCIBLE1
*LBD16*: LATERAL ORGAN BOUNDARIES DOMAIN16
GRAS: GAI-RGA-AND-SCR
*GSK3*: GLYCOGEN SYNTHASE KINASE3
*TIR1/AFB2*: TRANSPORT INHIBITOR RESPONSE1/AUXIN-SIGNALLING F-BOX2
*BZR1*: BRASSINAZOLE-RESISTANT1
*ERF*: ETHYLENE RESPONSE FACTOR115
*IPT*: ISOPENTENYL TRANSFERASES
ROS: Reactive oxygen species
mg/L: Milligram/Litre
mm: Millimetre
CD-HIT: Cluster database at high identity with tolerance
RSEM: RNA-seq by expectation maximization
FC: Fold-change
RNA-seq: RNA-sequencing
