# Supplementary material for: De novo transcriptome analysis provides insights into formation of in vitro adventitious root from leaf explants of Arnebia euchroma
Source: BMC Plant Biol. 2021 Sep 9;21:414. doi: 10.1186/s12870-021-03172-6 (PMC8427917; doi:10.1186/s12870-021-03172-6)
Supplement: Supplementary file 2 — Additional file 2 Fig. S2. Functional annotation with respect to adventitious root development in A. euchroma using GO enrichment analysis. [file 12870_2021_3172_MOESM2_ESM.docx]

***De novo* transcriptome analysis provides insights into formation of *in vitro* adventitious root from leaf explants of *Arnebia euchroma***

Jyoti Devi^1,2^, Ekjot Kaur^1,2^, Mohit Kumar Swarnkar^1^, Vishal Acharya^1,2^*, Shashi Bhushan^1,2,3*^

^1^Biotechnology Division, CSIR-Institute of Himalayan Bioresource Technology (IHBT), Palampur, H.P.-176061, India

^2^Academy of Scientific and Innovative Research (AcSIR), Ghaziabad-201002, India

^3^Dietetic & Nutrition Technology Division, CSIR-Institute of Himalayan Bioresource Technology (IHBT), Palampur, H.P.-176061, India

* Correspondence: sbhushan@ihbt.res.in & vishal@ihbt.res.in

**Fig. S2. Functional annotation with respect to adventitious root development in *A. euchroma* using GO enrichment analysis**

**Fig. S2a**

**Biological Process**

**
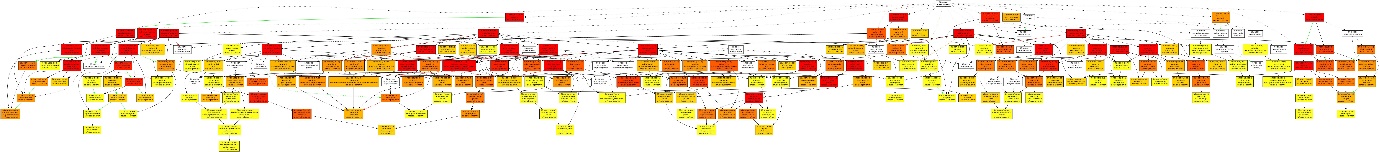
**

**Fig. S2b**

**Molecular function**


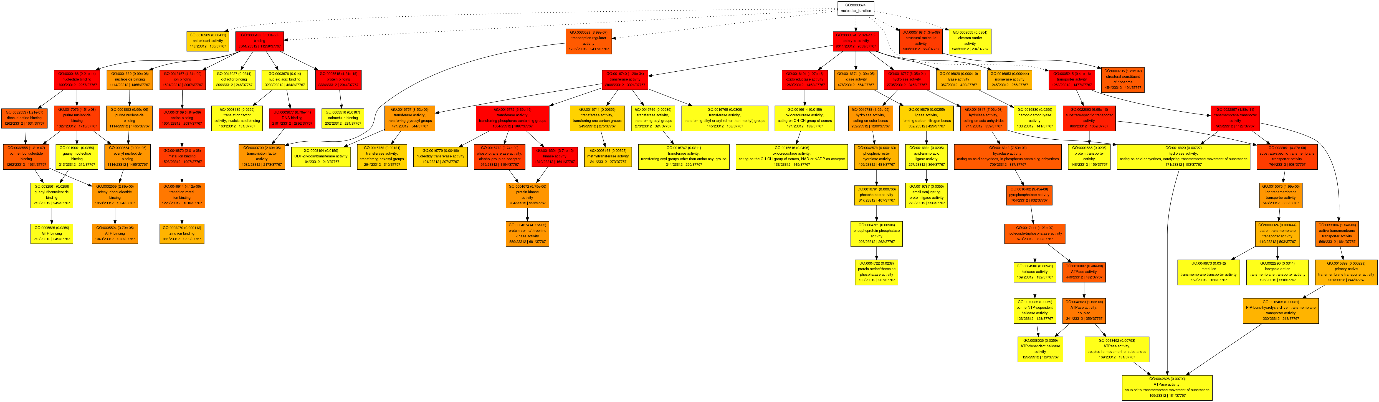


**Fig. S2c**

**Cellular Component**

**
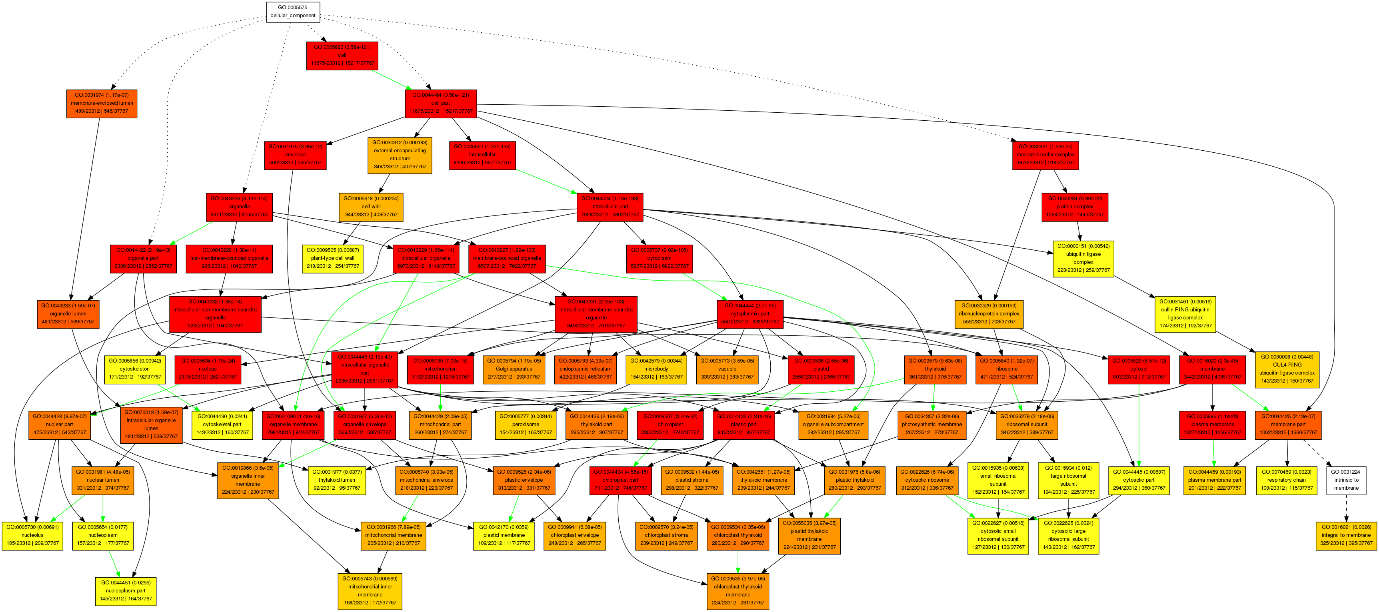
**
