# Supplementary material for: De novo transcriptome analysis provides insights into formation of in vitro adventitious root from leaf explants of Arnebia euchroma
Source: BMC Plant Biol. 2021 Sep 9;21:414. doi: 10.1186/s12870-021-03172-6 (PMC8427917; doi:10.1186/s12870-021-03172-6)
Supplement: Supplementary file 3 — Additional file 3 Table S1. Summary of de novo assembly of Arnebia euchroma leaf induced adventitious roots under in vitro conditions. [file 12870_2021_3172_MOESM3_ESM.docx]

***De novo* transcriptome analysis provides insights into formation of *in vitro* adventitious root from leaf explants of *Arnebia euchroma***

Jyoti Devi^1,2^, Ekjot Kaur^1,2^, Mohit Kumar Swarnkar^1^, Vishal Acharya^1,2*^, Shashi Bhushan^1,2,3*^

^1^Biotechnology Division, CSIR-Institute of Himalayan Bioresource Technology (IHBT), Palampur, H.P.-176061, India

^2^Academy of Scientific and Innovative Research (AcSIR), Ghaziabad-201002, India

^3^Dietetic & Nutrition Technology Division, CSIR-Institute of Himalayan Bioresource Technology (IHBT), Palampur, H.P.-176061, India

* Correspondence: sbhushan@ihbt.res.in & vishal@ihbt.res.in

**Table S1. Summary of *de novo* assembly of *Arnebia euchroma* leaf induced adventitious roots under *in vitro* conditions.**

|  | **Total number of unigenes** | **N50 (bps)** |
| --- | --- | --- |
| **Combined Raw Assembly / Non-filtered** | 3,21,502 | 1799 |
| **CPM>=10** **(CD-HIT-est)** | 1,62,420 | 1951 |
| **CPM>=20** **(CD-HIT-est)** | 1,29,804 | 2099 |
| **TPM>=0.5** **(CD-HIT-est)** | 54,587 | 2193 |
| **TPM>=1** **(CD-HIT-est)** | 39,523 | 2183 |
| **TPM>=2** **(CD-HIT-est)** | 29,086 | 2147 |
